# Subacute skin lesions in systemic lupus erythematosus: an impressive and well-tolerated response to anifrolumab^؆^

**DOI:** 10.1016/j.abd.2025.501220

**Published:** 2025-11-01

**Authors:** Bárbara Fernandes Esteves, Maria Seabra Rato, João Carlos Almeida, Miguel Bernardes, Lúcia Costa, Raquel Miriam Ferreira

**Affiliations:** a Department of Rheumatology, São João Local Health Unit, Hospital Center of São João, Porto, Portugal; b Department of Pathology, São João Local Health Unit, Hospital Center of São João, Porto, Portugal

Dear Editor,

Systemic lupus erythematosus (SLE) is a chronic multisystemic autoimmune disease with a wide spectrum of clinical manifestations.^1^ Muco-cutaneous lesions are common in SLE, affecting up to 80% of patients. Cutaneous lesions can cause significant morbidity and potentially lead to irreversible damage. When exposed to exogenous noxae (such as ultraviolet light), SLE patients’ skin presents enhanced susceptibility to cell damage. SLE skin has interferon (IFN) hyperresponsiveness to inflammatory stimuli, serving as a key site of autoantigen sensitisation, triggering systemic inflammation.^2^

The heterogeneity of clinical presentation of SLE contributes to its therapeutic challenge.^3^ This report presents an impressive and successful treatment outcome for cutaneous manifestations of SLE.

We describe the case of a 37-year-old woman with a one-year history of extensive erythematous annular lesions on the upper thorax, back, extensor surfaces of arms and forearms, gluteal region, and posterior upper thigh (Fig. 1) and non-scarring alopecia. The Cutaneous Lupus Erythematosus Disease Area and Severity Index activity (CLASI-A) score was 25, no damage (dyspigmentation or scarring) was observed. She also complained about inflammatory polyarthralgias, xerophthalmia and xerostomia. She had no relevant prior personal or family history. Laboratory tests revealed positivity for anti-SSA and anti-nuclear antibodies (titter of 1/1000; speckled pattern), with normal blood cell count, complement levels, and kidney function. Skin biopsy revealed histopathological findings (Fig. 2), as well as IgM deposition along the dermal epidermal junction on direct immunofluorescence, supporting the clinical diagnosis of subacute cutaneous lupus erythematosus. Schirmer’s test was normal, and the salivary gland biopsy didn’t reveal histopathological changes.

**Figure 1:**
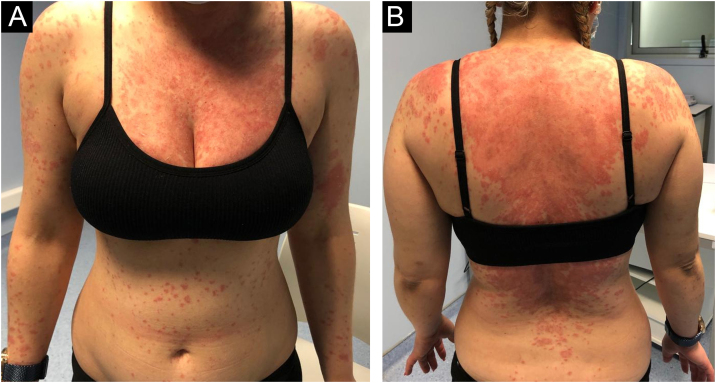
(A‒B) Subacute cutaneous lupus showing annular erythematous plaques coalescing into polycyclic plaques with raised borders and scaling before treatment.

**Figure 2:**
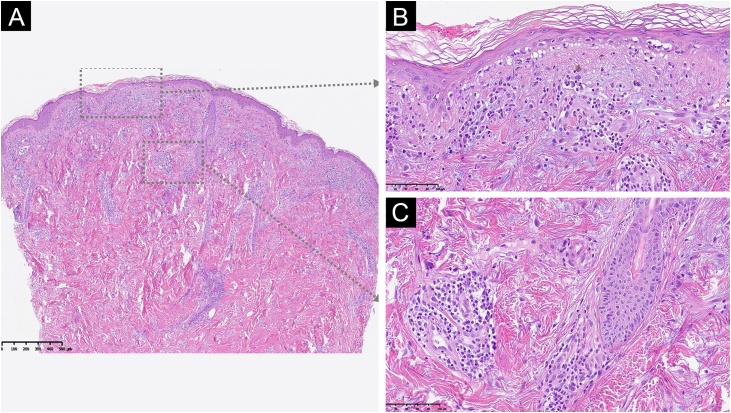
Morphological features consistent with the clinical diagnosis of subacute cutaneous lupus erythematosus subtype. Skin biopsy (Hemaxitolyn & eosin stain) showed epidermal thinning (A, ×40), areas of interface dermatitis with basal layer vacuolization (B, ×100), dermal mucin, pigment incontinence and perivascular lymphocytic infiltrate (C, ×100).

A diagnosis of SLE was established based on the 2019 EULAR/ACR Classification Criteria for Systemic Lupus Erythematosus, and hydroxychloroquine was initiated. However, only improvement of the articular involvement was observed. For that reason, oral prednisolone up to 40 mg/day, topical betamethasone, and azathioprine up to 1.5 mg/kg/day were introduced, without response. Azathioprine was discontinued due to severe lymphopenia. Mycophenolate mofetil 1500 mg/day was tried, but lymphopenia was once again observed. The iatrogenic effect was confirmed systematically with normalization of white cell count after drug withdrawal.

Given the patient’s significant disease activity despite multimodal therapy, anifrolumab 300 mg infusion was started every 4-weeks. One month later, the patient showed a dramatic improvement of the skin lesions (Fig. 3) and the non-scarring alopecia with a CLASI-A score drop to 7. The dose of prednisolone was progressively tapered to 5 mg/day in three months. No adverse effects have been noted during biologic therapy.

**Figure 3:**
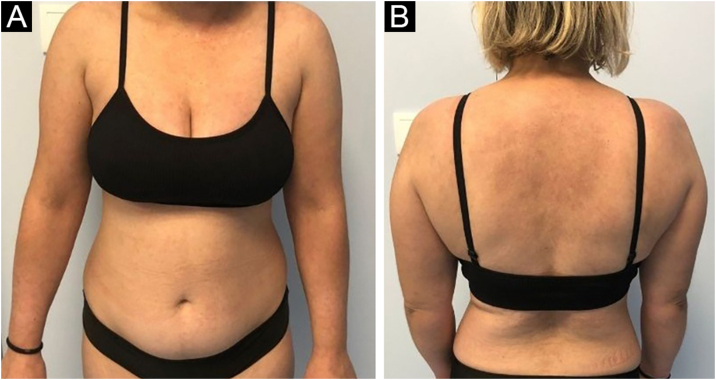
(A‒B) Improvement after two infusions of anifrolumab with total resolution of the lesions.

Activation of the type I IFN pathway is recognized as playing a critical role in the pathogenesis of SLE, promoting dendritic cell formation, activation of T-cells, and autoantibodies production by B-cells.^1^, ^4^, ^5^ Anifrolumab, a fully human monoclonal antibody that selectively binds to IFN receptor subunit 1, inhibits signaling by all type I interferons.^3^, ^5^, ^6^ This blockade may reverse immune dysregulation and prevent further tissue damage in SLE.^4^

Anifrolumab was recently approved for the treatment of moderate-severe SLE in addition to the standard care for patients without renal or CNS involvement.^4^, ^5^, ^7^ The main three anifrolumab trials (MUSE,^5^ TULIP-1^8^ and TULIP-2^3^) evaluated cutaneous manifestation response using the CLASI score. All trials showed a rapid significant reduction in CLASI activity with anifrolumab. Sustained oral prednisolone tapering until dosage ≤7.5 mg/day was also achieved.^3^, ^5^, ^7^, ^8^ Another important observation was the rapid skin response, which can impact daily life, since the most commonly involved areas are exposed and visible.^2^, ^6^

This case highlights the value of anifrolumab in the treatment of cutaneous lupus after the failure of multiple therapies, due to ineffectiveness or adverse effects.

## ORCID IDs

Maria Seabra Rato: 0000-0002-5037-1016

João Carlos Almeida: 0009-0002-5402-6217

Miguel Bernardes: 0000-0002-1849-5465

Lúcia Costa: 0000-0002-5817-5394

Raquel Miriam Ferreira: 0000-0001-8527-7508

## Financial support

The authors report that there is no funding associated with the work featured in this article.

## Authors’ contributions

Bárbara Fernandes Esteves: Data collection, or analysis and interpretation of data; Writing of the manuscript or critical review of important intellectual content; Critical review of the literature; Final approval of the final version of the manuscript.

Maria Seabra Rato: Data collection, or analysis and interpretation of data; Writing of the manuscript or critical review of important intellectual content.

João Carlos Almeida: Data collection, analysis, and interpretation.

Miguel Bernardes: Final approval of the final version of the manuscript.

Lúcia Costa: Final approval of the final version of the manuscript.

Raquel Miriam Ferreira: The study concept and design; writing of the manuscript or critical review of important intellectual content; Final approval of the final version of the manuscript.

## Research data availability

Does not apply.

## Conflicts of interest

Miguel Bernardes: Advisor board - Lilly, Biogen, Abbvie, Pfizer, Novartis, Boehringer Ingelheim BMS, AstraZeneca, Janssen-Cilag. Speaker - Lilly, Abbvie, Pfizer, Boehringer Ingelheim, AstraZeneca, Janssen-Cilag. Honorary compensation - Lilly, Biogen, Abbvie, Pfizer, Novartis, Boehringer Ingelheim BMS, AstraZeneca, Janssen-Cilag.

No potential conflict of interest was reported by the other authors.
